# Challenges in evaluating thromboembolic risk in acute leukemia: clinical profiling and limitations of current scoring systems

**DOI:** 10.1016/j.rpth.2025.103017

**Published:** 2025-08-19

**Authors:** Luisa V. Carvalho, José Vanildo R. de Oliveira, Raphael B. Melo, Fernanda R. Mendes, Cynthia Rothschild, Elvira D.R.P. Velloso, Vanderson Rocha, Eduardo M. Rego, Fernanda A. Orsi, Wellington F. Silva

**Affiliations:** 1 Division of Hematology, Hospital das Clínicas da Faculdade de Medicina da Universidade de São Paulo, São Paulo, Brazil; 2 Department of Pathology, School of Medical Sciences, Unicamp, Campinas, Brazil

**Keywords:** acute leukemia, arterial thrombosis, disseminated intravascular coagulation, clinical decision rules, venous thrombosis

## Abstract

**Background:**

Limited evidence is available on risk factors for thrombotic events in newly diagnosed acute leukemia (AL) patients, and predictive tools in this population need further validation.

**Objectives:**

To evaluate the incidence of thrombosis in newly diagnosed AL patients and to validate known predictive scores.

**Methods:**

We retrospectively included 421 patients between 2009 and 2022. Data on thrombotic events (venous and arterial) were collected from admission to 60 days of follow-up. The Khorana, International Society on Thrombosis and Haemostasis (ISTH) disseminated intravascular coagulation, and Siriraj Acute Myeloid/Lymphoblastic Leukemia scores were applied to the cohort.

**Results:**

The cumulative incidence of thrombotic events within 60 days was 15.8%, being higher in the acute lymphoblastic leukemia subgroup (21%). Seventy-two percent of the events occurred within the first 30 days of diagnosis. Superficial vein thrombosis and catheter-related thrombosis comprised most events (38.2% and 19.1%, respectively), while arterial thrombosis represented 11.8%. Obesity and platelet count > 20 × 10^9^/L were associated with an overall risk of thrombosis in this cohort. In the acute myeloid leukemia subset, thrombotic event occurrence was associated with a higher peripheral blast percentage and elevated C-reactive protein levels. The Khorana, ISTH-disseminated intravascular coagulation, and Siriraj Acute Myeloid/Lymphoblastic Leukemia scores were not associated with thrombotic events in AL patients.

**Conclusion:**

The incidence of thrombotic events is not negligible, being higher in the induction phase. Further studies and better tools to improve event prediction in this population, such as biomarker development, are needed.

## Essentials

• Thrombotic risk in AL patients remains incompletely understood.
• In 421 patients with AL, 15.8% developed TEs within 60 days of diagnosis.
• Most events were related to a central venous catheter or superficial vein thrombosis.
• Current risk scores failed to predict thrombotic events in AL, and better tools are needed.

## 1 Introduction

Patients with newly diagnosed acute leukemia (AL) have an increased risk of thrombotic events (TEs). The reported 1-year cumulative incidence of leukemia-associated thrombosis ranges from 2% to 25% [1]. Leukemia subtype influences the risk of thrombosis, which is probably higher in acute promyelocytic leukemia (APL) at 20.6% during induction, based on previous retrospective analysis [2]. In a Brazilian cohort of acute myeloid leukemia (AML) patients, the described incidence was 11.9% [3].

The increased thrombotic risk in AL is the result of several mechanisms, including factors related to the disease itself, the treatment administered, and the patient’s comorbidities and history. AL blast cells can induce a hypercoagulable state through the expression of tissue factor, the release of procoagulant microvesicles, and the activation of platelets and endothelial cells [4,5]. AL treatment can also lead to endothelial injury, and asparaginase reduces the levels of natural anticoagulants, such as protein C and antithrombin [6]. Established risk factors for arterial and venous thrombosis (VT), such as age and cardiovascular risk factors (eg, arterial hypertension, diabetes, dyslipidemia, and obesity), have also been proposed as independent factors for thrombosis in the AL population [1,2,7]. However, studies addressing these variables are scarce and heterogeneous, with inconsistent findings. Inconsistencies also emerge when correlating the results of routine coagulation assays, such as thrombocytopenia, changes in prothrombin time (PT), activated partial thromboplastin time, and fibrinogen levels, with the risk of thrombosis. D-dimer, a marker for fibrinolysis, has been shown to predict venous and arterial thrombosis (AT) in AML and acute lymphoblastic leukemia (ALL) [[8], [9], [10]], but not in APL [[11], [12], [13]].

Predicting the occurrence of thrombosis in AL is still a matter of debate. The Khorana score can predict symptomatic venous thromboembolism in outpatients with solid cancer [14]. However, it was unable to effectively predict thrombosis in patients with AL [15]. The ISTH score for disseminated intravascular coagulation (DIC) has been shown to be an effective tool for predicting thrombosis and bleeding events, but only in specific subsets of leukemia [16,17]. More recently, the Siriraj Acute Myeloid/Lymphoblastic Leukemia (SiAML) score was also developed to identify those at a higher risk of thrombosis complications in AL [9]. Additional validation of the ISTH-DIC and SiAML scores is required before they can be routinely used in a clinical setting.

Even though the impact of thrombosis on AL survival has not been demonstrated [4], the occurrence of TEs in this population can lead to prolonged hospitalization, potential central nervous system complications [18], and treatment delay. In this context, predicting the risk of thrombosis in AL patients is crucial to delineate strategies to prevent this complication. Therefore, this study aimed to describe the incidence of TEs in a Brazilian cohort, to identify clinical and laboratory factors that can predict thrombosis in AL patients, as well as to validate prior scores and report their clinical profiles and associated findings related to these events.

## 2 Methods

### 2.1 Patients and study design

This was a retrospective cohort analysis of patients aged >15 years with newly diagnosed AL admitted to a reference cancer center in Brazil between 2009 and 2022. Patients with AL were included regardless of phenotype (ALL, AML, or APL). Subjects with lymphoblastic lymphoma without bone marrow infiltration were excluded. Patients were followed up from the date of diagnosis to 60 days posttreatment initiation. Therapeutic protocols were reported elsewhere [[19], [20], [21]]. We divided treatment into intensive and nonintensive for ALL and AML patients, and we assumed intensive treatment for all APL subjects.

At our center, all patients diagnosed with AML who are eligible for intensive treatment undergo central line catheter placement, while for patients with APL or ALL, the decision is left to the physician’s discretion. Antithrombotic prophylaxis was given with low-molecular-weight heparin for inpatients with platelet counts > 50 × 10^9^/L. For those with platelet counts between 25 and 50 × 10^9^/L, use of thromboprophylaxis was individualized based on clinical assessment [22]. Demographic, clinical, and laboratory parameters at the time of diagnosis, including age, sex, body mass index (BMI), contraceptive use, white blood cell count (WBC), hemoglobin level, platelet counts, PT, activated partial thromboplastin time, D-dimer, and fibrinogen, were retrieved from electronic medical charts. For patients with a TE, clinical and laboratory data on hemoglobin level, differential WBC, and platelet count at the time of the event were also collected.

This study was approved by the Research Ethics Board of the University of São Paulo (CAAE 80531817.3.0000.0068).

### 2.2 Study definitions and procedures

The primary endpoint was the cumulative incidence of any TE. TE was a composite endpoint defined as the occurrence of VT, AT, catheter-related thrombosis (CRT), and superficial vein thrombosis (SVT) within the first 60 days following diagnosis. CRT was defined as symptomatic deep vein thrombosis associated with a central venous catheter. We considered a central venous catheter to be a catheter that extends into the superior vena cava or femoral vein [23]. SVT was defined as any symptomatic thrombosis in a superficial vein [24]. VT was defined as a symptomatic thrombosis, not related to an inserted catheter, in the upper (axillary, subclavian, and/or jugular veins) or lower extremities (femoral, popliteal, superficial femoral, and/or iliac veins), pulmonary embolism, splanchnic vein thrombosis, and cerebral VT (CVT). VT had to be present clinically and supported by imaging studies, which included Doppler ultrasonography or computed tomography angiography. AT included myocardial infarction, acute arterial occlusion, and ischemic stroke. Death was considered a competing event for thrombotic outcomes.

Secondary endpoint included the risk of VT or AT within 60 days following diagnosis, which are the TEs most associated with increased morbidity and mortality.

The Khorana score was calculated based on BMI, and leucocyte, hemoglobin, and platelet count at admission [14]. The ISTH-DIC score was calculated with values of D-dimer, PT, platelet count, and fibrinogen level. D-dimer was measured through immunoturbidimetry, and we used a cutoff of 5000 ng/mL to define a significant increase. We considered a score of ≥5 points (“positive”) as suggestive of DIC according to the original publication [25]. The SiAML score was based on WBC, platelet counts, and D-dimer [9]. All predictive scores were calculated using the admission parameters prior to the start of any treatment. Missing data for parameters included in the scores were assessed using Little’s missing completely at random test [26], which showed no significant deviation from randomness (*P* = .617), suggesting the data were missing completely at random. Based on this, listwise deletion was deemed appropriate.

### 2.3 Statistical analysis

Categorical variables were presented as numbers and percentages, while numerical variables were presented as mean and SD for normally distributed variables and median and IQR for nonnormally distributed variables. Overall survival was estimated by the Kaplan–Meier method. Univariate comparisons between groups were evaluated using the chi-square test or Fisher’s exact test for categorical data or the Mann–Whitney *U*-test or median test for continuous data. Cumulative incidences were calculated by competing risk analysis. A multivariate logistic regression model was used to identify risk factors associated with the primary endpoint. We included adjusted variables with a *P* value < .2 in the univariate analysis or previously reported to be related to the outcome in the multivariable model. The cutoff for numerical variables was defined by the Youden index method as appropriate. Associations between risk scores and thrombosis were investigated after categorization through chi-squared test or Fisher’s exact test.

## 3 Results

A total of 421 patients were included (see Supplementary Figure). Most patients were men (51.5%), with a median age at diagnosis of 45 years (range, 15-79). Obesity was registered in 19.5% of patients (BMI ≥ 30 kg/m^2^). Baseline diagnosis were AML, ALL, and APL in 49%, 28%, and 23% of the patients, respectively. Median WBC was 16.6 × 10^9^/L (range, 0.26-580 × 10^9^/L). Baseline characteristics are summarized in Table 1.

**Table 1:** Baseline characteristics of the cohort.

Variables	*N* = 423
Male sex, *n* (%)	218 (51.5)
Age (y), median (range)	45 (15-79)
BMI (kg/m^2^), median (range)	25 (13-48.3)
Subtype, *n* (%)	
ALL	120 (28.4)
AML	208 (49.2)
APL	95 (22.4)
Monocytic AML, *n* (%)	100 (48.3)
ALL subtype, *n* (%)	
B Ph negative	56 (13.3)
B Ph positive	34 (8.0)
T ALL	25 (6.0)
MPAL	5 (1.2)
Use of asparaginase, *n* (%)	47 (47.9)

Laboratory variables	Median (range)
Hemoglobin (g/dL)	7.7 (3.1-15.8)
WBC (×10^9^/L)	16.6 (0.26-580)
Peripheral blasts (%)	52.5 (0-98)
Platelets (×10^9^/L)	30 (1-580)
Fibrinogen (IU/L)	303 (50-909)
D-dimer (ng/mL)	5859 (80-128,000)
C-reactive protein (g/dL)	44.7 (0.5-550)
PT (s)	15.5 (7.4-95)
Partial activated thromboplastin time (ratio)	1 (0.7-3.1)

ALL, acute lymphoblastic leukemia; AML, acute myeloid leukemia; APL, acute promyelocytic leukemia; BMI, body mass index; MPAL, mixed phenotype acute leucemia; PT, prothrombin time; T ALL, T acute lymphoblastic leukemia; WBC, white blood cell count.

The overall 60-day cumulative incidence of a TE (including AT, VT, CRT, and SVT) was 15.8% (95% CI, 12%-18%), with most events occurring in ALL and APL patients. When excluding SVT and CRT from the analysis, there were 21 events of VT or AT, with a 60-day cumulative incidence of 4.9%.

For each leukemia subgroup, the 60-day cumulative incidence of a TE was 21%, 20%, and 11% in ALL, APL, and AML, respectively (*P* = .03). Most events occurred within the first 30 days of diagnosis (72%), while the remaining cases were detected at the time of disease diagnosis (24%) or between 30 and 60 days after the diagnosis (4%).

**Figure 1:**
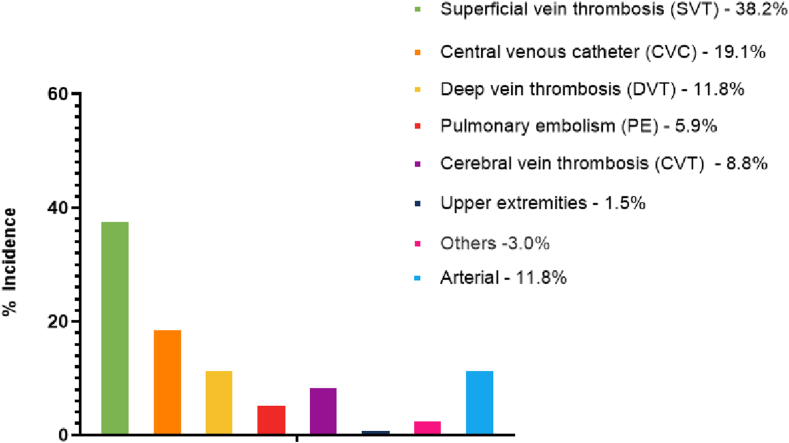
Cumulative incidence of thrombotic events per site.

**Figure 2:**
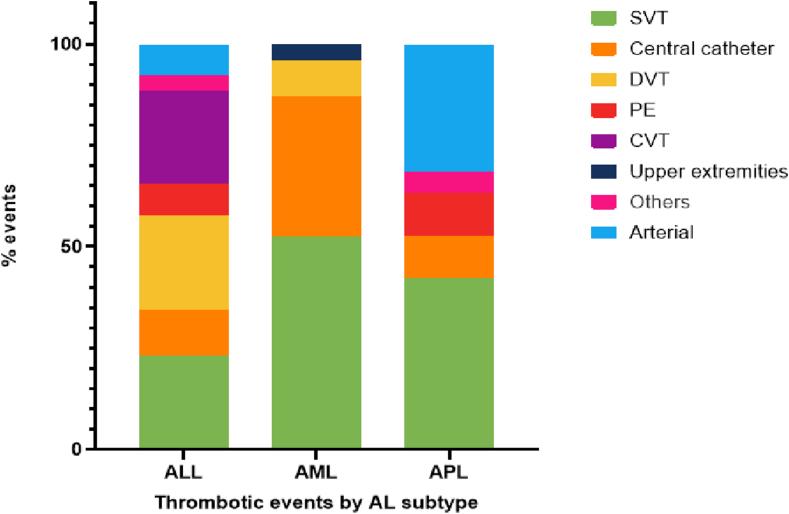
Thrombotic events according to acute leukemia subtype. ALL, acute lymphoblastic leukemia; AML, acute myeloid leukemia; APL, acute promyelocytic leukemia; CVT, cerebral vein thrombosis; DVT, deep vein thrombosis; PE, pulmonary embolism; SVT, superficial vein thrombosis.

The most common TE was SVT of the upper extremity (38.2%), followed by CRT (19.1%), deep vein thrombosis of the lower extremity (11.8%), and pulmonary embolism (5.9%; see Figure 1). When analyzing the type of thrombosis by AL subgroup, SVT represented the majority of events in AML (52.2%) and APL (42.1%), while CVT represented 23% of the events in ALL (Figure 2).

AT comprised 8 out of 68 TEs (11.8%), all of which were cerebrovascular (Figure 2). These were more frequent in APL (31.5%), which included 3 cases diagnosed at the initial presentation. One patient presented with concomitant VT and AT. Five patients (7.4%) experienced recurrent thrombosis during the 60-day period of follow-up.

Among those with AL and thrombosis, prophylactic anticoagulation was being administered at the time of thrombosis in 14.5% of patients. Two patients were receiving combined estrogen-progesterone therapy at admission, while 20% were using a progesterone-only contraceptive. Overall, 64.6% of patients with thrombosis had a concomitant active infection at the time of the event. Additionally, 33% of subjects still exhibited blasts in the peripheral blood, and 25% had platelet counts < 30 × 10^9^/L.

Univariate analysis of all TE and VT events, excluding SVT and CRT, was performed (Supplementary Tables 1 and 2). In a multivariable analysis of all TEs (including SVT and CRT), we found that obesity and platelet count > 20 × 10^9^/L were associated with an overall risk of thrombosis in our cohort (Table 2).

**Table 2:** Multivariable analysis for all thrombotic events in the whole cohort.

Variable	OR	95% CI	*P* value
Obesity	1.99	1.04-3.73	.035
Leukemia subtype	ALL: reference		
	AML: 0.75	0.39-1.42	.52
	APL: 1.30	0.63-2.65	.47
Platelets ≥ 20 × 10^9^/L	2.45	1.26-5.05	.01

ALL, acute lymphoblastic leukemia; AML, acute myeloid leukemia; APL, acute promyelocytic leukemia; OR, odds ratio.

Among patients with ALL, 40% (10/25) of TEs occurred in association with asparaginase use. For exploratory purposes, we conducted a univariate analysis in this subgroup (Supplementary Table 4), which showed an association between TEs and higher platelet counts (mean platelet count, 52.8 vs 91.6 × 10^9^/L; *P* = .02). In the AML subset, TE occurrence was associated with a higher peripheral blast percentage (41.4% vs 57.2%; *P* = .026) and elevated baseline C-reactive protein levels (65.97 vs 103.82 mg/L; *P* = .04; Supplementary Table 5). No significant associations were found in the APL subgroup in this univariate analysis.

When excluding SVT and CRT, the parameters that independently associated with VT and AT were a diagnosis of ALL (*P* < .001) and younger age (*P* = .04), while platelet counts above 20 × 10^9^/L (*P* = .09) and male sex (*P* = .09) were marginally associated with these events (Supplementary Table 2). Multivariable analysis was not performed due to the low number of events unrelated to catheter placement.

### 3.1 Khorana, DIC, and SiAML scores

The Khorana score (*n* = 327) was low, intermediate, and high in 5.2%, 92.4%, and 2.4% of cases, respectively, with no statistical association with thrombosis in univariate analysis (*P* = .42; Table 3).

**Table 3:** Univariate analysis of predictive scores for any thrombotic events.

Score	Without TE	With TE	*P* value
**Khorana score (*n* = 327), *n* (%)**			.42
Low	13 (4.9)	4 (6.1)	
Intermediate	245 (93.2)	59 (89.4)	
High	5 (1.9)	3 (4.5)	
**DIC score ≥5 (*n* = 227), *n* (%)**			.785
	165 (87.8)	33 (84.6)	
**Median DIC (*n* = 227), mean (SD)**			.817
	5.80 (1.34)	5.74 (1.29)	
**SiAML high risk (*n* = 246), *n* (%)**			.709
	16 (7.8)	2 (4.8)	

DIC, disseminated intravascular coagulation; SiAML, Siriraj Acute Myeloid/Lymphoblastic Leukemia; TE, thrombotic event.

The ISTH-DIC score (*n* = 227) was positive at diagnosis in 87.2% of the cohort and was not associated with the occurrence of thrombosis (*P* = .785; Table 3). A similar result was observed when excluding patients with SVT and CRT, though this subanalysis should be interpreted with caution due to the low number of events (Supplementary Table 3).

The SiAML thrombosis score (*n* = 246), which includes distinct cutoffs for WBC, platelets, and D-dimer to stratify the risk of thrombosis in AML, was also not associated with thrombosis in our population (*P* = .709; Table 3).

## 4 Discussion

In this Brazilian cohort of 421 AL patients, the overall 60-day cumulative incidence of TEs was 15.8%. A high incidence of SVT was observed, accounting for 38.2% of the events, followed by CRT (19.1%), which is in line with previously reported results [1,[27], [28], [29]]. Nevertheless, most studies represent populations from North America and Europe, whereas populations from Latin America are less frequently studied. From a global perspective, our results confirm that AL is associated with a substantial risk of thrombosis in an otherwise underrepresented population.

In a US cohort of 299 patients with ALL and 996 with AML, the cumulative incidence of venous thromboembolism was 17.7% and 8.6%, respectively, with most events occurring in the upper extremities (either central venous catheter-related or not) [1]. Similarly, in a Canadian cohort of 501 AL patients (427 with AML and 74 with ALL), the cumulative incidence of thrombosis was 15.3%, with CRT representing 43.5% of the events [30].

After excluding the cases of SVT and CRT, we found a 60-day cumulative incidence of AT and VT of 4.9% (21/421). Of note, in a US cohort of AML, the 2-year cumulative incidence of VT was similar (5.2%) [31].

Even though SVT is not associated with an increased risk of PE or death, such as VT and AT, it poses a relevant clinical impact on AL patients [32]. Symptomatic SVT events, such as those included in this study, require medical intervention, including antibiotic therapy, anticoagulation (if feasible), or both. In the context of AL treatment, where neutropenia, infections, and thrombocytopenia are expected, the use of antibiotics or anticoagulants may exacerbate the risk of multidrug-resistant or fungal infections, while anticoagulation increases the risk of bleeding. From this perspective, SVT might represent a more severe complication in patients with AL than in those with solid cancer or other diseases [33].

In this analysis, ALL was independently associated with an increased risk of TEs in our study. The 60-day cumulative incidence of thrombosis in ALL was 21%, consistent with the previously reported incidence range of 16% to 24.5% in the literature [10,34]. In the current study, asparaginase played a role in part of these TEs, even though the exact interaction with the disease itself is unclear. There were 6 CVT events in the ALL cohort, corresponding to an overall incidence of 5%, in line with previous reports of 3% CVT incidence [18]. A prior study by our group has reported a similar incidence after PEG-asparaginase [35].

In the multivariable analysis, obesity and platelet levels above 20 × 10^9^/L were independently associated with TEs in patients with AL. Obesity appeared to be more strongly associated with thrombosis in ALL patients, consistent with prior reports [36,37]. In AML, TEs were associated with peripheral blast burden and elevated C-reactive protein levels at presentation. These findings suggest that thrombosis in AML may be more closely related to the severity of illness at diagnosis, as more critically ill patients are often immobilized and undergo early catheter placement.

Additionally, we attempted to validate 3 clinical prediction scores for thrombosis in cancer patients but were unable to demonstrate any significant associations in our cohort. We recognize that the scoring systems we applied were originally validated in distinct populations. The Khorana score did not include AL patients in its development cohort, and prior validation attempts in this population have demonstrated its limited ability to predict thrombosis [8,15]. Our results align with those previously published [8,15]. Possibly, the main factor that impairs the applicability of Khorana score in AL patients is the high platelet threshold of 350 × 10^9^/L, rarely found in this disease.

Similarly, the ISTH-DIC score was not predictive of thrombosis in AL. In the literature, there is available information on its application to cohorts of patients with AML [8,16]. The different mechanisms contributing to thrombosis in other subtypes of AL and missing data on D-dimer in part of the cohort might have influenced our results. Even though the ISTH-DIC score was used to predict VT and AT in the mentioned studies [8,16], they did not include catheter-related events. However, even when we excluded CRT and SVT from the analysis, it remained nonpredictive of thrombosis in our cohort. Previous studies have reported conflicting results regarding the accuracy of the ISTH-DIC tool in predicting bleeding or TEs in AL [8,9,38], with some studies reporting positive results depending on the cutoff applied [16,17]. Calculation of the ISTH-DIC score based on parameters at diagnosis could have affected the results, as chemotherapy influences thromboinflammatory mechanisms, and coagulation tests change after therapy. The SiAML score, which originally did not account for CRT, was also not helpful in predicting thrombosis in our cohort, and further validation is needed to assess its performance.

There are some aspects of this study that need to be highlighted. Its retrospective nature precluded us from analyzing important known risk factors for TEs that were not thoroughly registered in all medical charts, such as a previous history of thrombosis, comorbidities, or smoking. After excluding SVT and CRT, the small number of remaining events rendered the univariate analysis underpowered, preventing the performance of multivariate analysis and subanalysis of risk factors for thrombosis in each AL subtype. We also acknowledge that analyzing all AL subtypes together is not ideal, given the distinct pathophysiological pathways underlying TEs, whether related to the disease itself or to treatment protocols.

Nevertheless, this study presents data from a large, real-world cohort treated at a reference center and, to our knowledge, is the first to describe thrombosis in patients with AL from Latin America (Table 4). TEs were observed even in the presence of thrombocytopenia, prolonged PT, or hypofibrinogenemia, suggesting a complex coagulopathy that may lead to a hypercoagulable state in AL. However, these parameters do not appear to reliably predict thrombosis, and currently available risk scores have shown limited utility. Thus, the identification of novel coagulation biomarkers, particularly those reflecting global coagulation activity or thromboinflammatory processes, is warranted to improve the understanding of both thrombotic and bleeding risks in this patient population.

**Table 4:** Selected studies on thromboembolic events in acute leukemia patients.

Study	*N*	Thrombosis incidence (%)	Type of thrombosis	Platelet count at the time of thrombosis (×10^9^/L)
Ku et al. [31] USA	• 5394: AML • 2483: ALL (51% < 15 y)	AML: 282 events • 3-mo C.I: 3.3% ALL: 113 events • 3-mo C.I: 2.5%	Only VT/CRT were included.	NA
Vu et al. [1] USA	• 299: ALL • 923: AML • 73: APL	10.7% (139 events) • 17.7% ALL • 8.6% AML • 11% APL	• CRT: 53% ALL/61.6% AML • SVT: NA • VT: 47% ALL/38% AML • AT: NA	49.6% of patients in the range of 51 to 99.
Mitrovic et al. [2] Serbia	• 63: APL	20.6% (13 events)	• CRT: 3/13 (23%) • SVT: NA • VT: 6/13 (46.1%) • AT: 4/13 (30%)	NA
Libourel et al. [16] Netherlands	• 272: AML	8.7% (24 events)	• CRT: NA • SVT: NA • VT: 4.7% of the whole cohort • AT: 4% of the whole cohort	Not significantly associated with thrombosis.
Al-Ani et al. [30] Canada	• 74: ALL • 386: AML • 41: APL	15.3% (77 events) 3-mo C.I: 9.6% • 49 events AML • 28 events ALL	• CRT: 42/77 (54.5%) • SVT: NA • VT: 35/77 (45.4%) • AT: NA	>50: associated with increased thrombotic risk.
Xiao et al. [13] China	• 83: APL (18% < 21 y)	14.4% (12 events)	• CRT: NA • SVT: NA • VT: 5/12 • AT: 5/12	Not significantly associated with thrombosis.
Anderson et al. [10] USA	• 61: ALL	• 100-d C.I: 24.7% (15 events)	• CRT: 4/15 (27%) • SVT: not included • VT: 7/15 (46%) • AT: 4/15 (27%)	NA
Martella et al. [8] Italy	• 210: AML • 12: high-risk MDS	22.1% (50 events) • 6-mo C.I: 10%	• CRT: 28/50 (56%) • SVT: NA • VT: 17/50 (34%) • AT: 5/50 (10%)	63% with platelets >50.
Paterno et al. [38] Italy	• 300: AML	14.6% (44 events) • 45-d C.I: 9.2% • 3-mo C.I: 11.1%	• CRT: 28/44 (63.6%) • SVT: not included • VT: 9/44 (20.4%) • AT: 7/44 (15.9%)	Platelets >50: OR, 3.28 for VT/CRT (95% CI, 1.41-7.64; *P* = .006).
Hellou et al. [27] Israel	• 335: AML	9.8% (33 events)	• CRT: 28/33 (85%) • SVT: NA • VT: 5/33 (15%) • AT: NA	Not significantly associated with thrombosis.
Shimony et al. [37] USA	• 191: ALL (Dana Farber cohort)	36.1% (82 events) • 1-mo C.I: 9.9% • 6-mo C.I: 24.2%	• CRT/SVT 20/82 (24.4%) • VT: 62/82 (75.6%) • AT: NA	NA
Hisada et al. [29] USA	• 76: ALL • 253: AML • 29: APL	10.3% (37 events) 1-y C.I: 11.3% • 7.3% ALL • 12.9% AML 10.4% APL	• CRT: 15/37 (40.5%) • SVT: 13/37 (35.1%) • VT: 9/27 (24.3%) • AT: NA	Not significantly associated with thrombosis.

ALL, acute lymphoblastic leukemia; AML, acute myeloid leukemia; APL, acute promyelocytic leukemia; AT, arterial thrombosis; C.I, cumulative incidence; CRT, catheter-related thrombosis; MDS, myelodisplastic syndrome; NA, not available; OR, odds ratio; SVT, superficial vein thrombosis; VT, venous thrombosis.
