# Supplementary material for: Challenges in evaluating thromboembolic risk in acute leukemia: clinical profiling and limitations of current scoring systems
Source: Res Pract Thromb Haemost. 2025 Aug 19;9(6):103017. doi: 10.1016/j.rpth.2025.103017 (PMC12455106; doi:10.1016/j.rpth.2025.103017)
Supplement: Supplementary Figure and Tables [file mmc1.docx]

***Figure S1:*** *Flow diagram illustrating cohort selection. Acute lymphoblastic leukemia (ALL); Acute myeloid leukemia (AML); Acute promyelocytic leukemia (APL). * Other thrombosis included splanchnic vein thrombosis.*


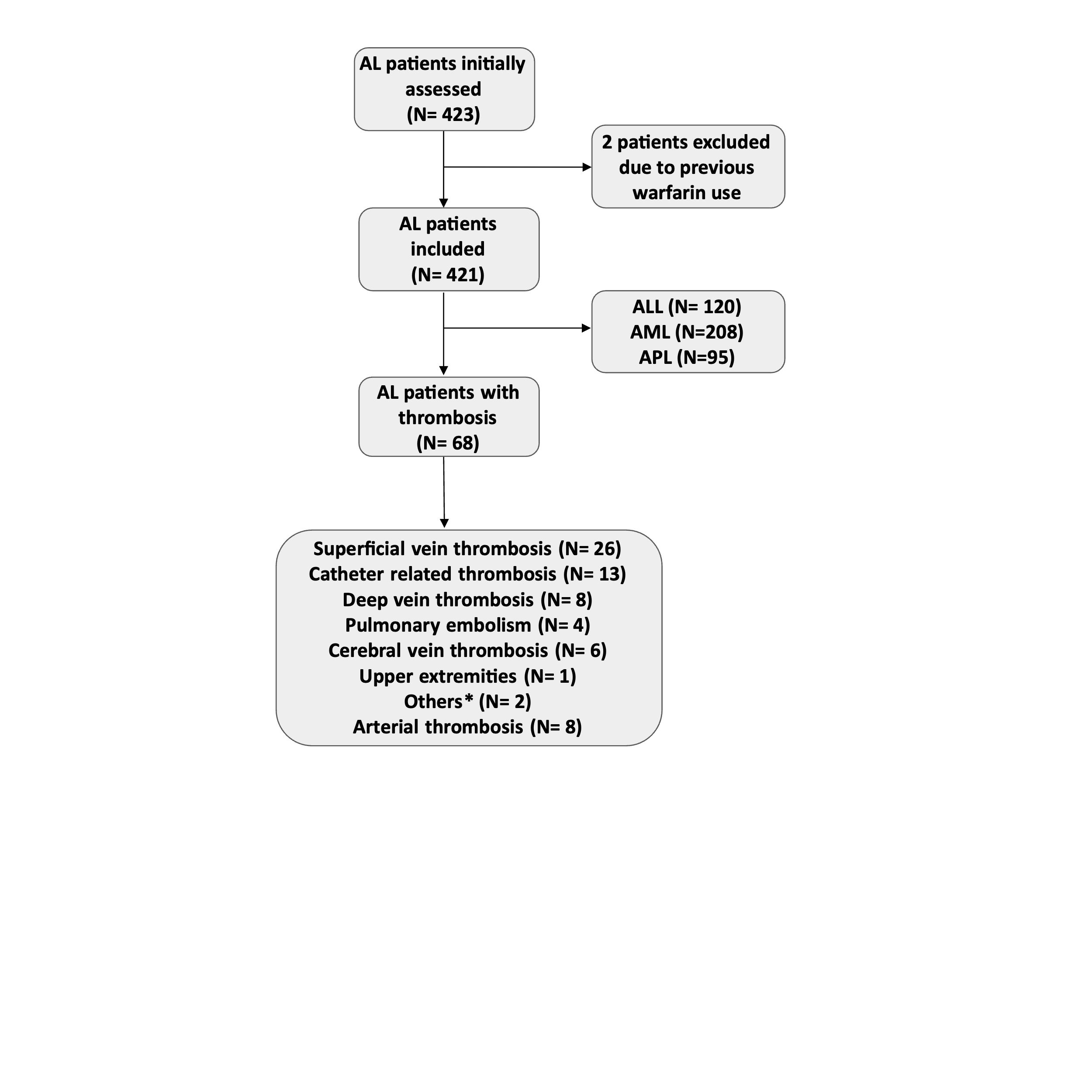


***Table S1. Supplementary Appendix:*** *Univariate analysis of risk factors for thrombotic events (TE) at 60 days*

| Variable | OR | CI 95% | p-value |
| --- | --- | --- | --- |
| Male sex | 0.84 | 0.5–1.43 | 0.527 |
| BMI (numerical) | 1.03 | 0.98–1.08 | 0.284 |
| Obesity | 1.94 | 1.03–3.58 | 0.037 |
| Age (numerical) | 0.99 | 0.98–1.01 | 0.401 |
| Leukemia subtype | ALL: reference  AML: 0.48  APL: 0.95 | 0.26–0.89  0.48–1.85 | 0.019  0.88 |
| White blood cell count (x10^9^/L) | 1 | 1–1 | 0.543 |
| Peripheral blasts (%) | 1 | 0.99–1.01 | 0.455 |
| Peripheral blood monocytes (%) | 1 | 1–1 | 0.739 |
| Platelets ≥20x10^9^/L | 2.16 | 1.17–4.27 | 0.019 |
| Prothrombine time ratio | 0.98 | 0.65–1.23 | 0.898 |
| CRP | 1 | 1–1 | 0.308 |
| Use of asparaginase  (ALL only) | 1.11 | 0.43–2.9 | 0.828 |
| Intensive treatment | 0.93 | 0.21–2.87 | 0.909 |

*OR: Odds-ratio; CI: confidence interval; BMI: body mass index; CRP: C-reactive protein.

***Table S2. Supplementary Appendix.*** *Univariate analysis of risk factors for venous thrombosis (VT) at 60 days* *(excluding SVT and CRT).*

| Variable | OR | CI 95% | p-value |
| --- | --- | --- | --- |
| Male sex | 0.46 | 0.17–1.12 | 0.098 |
| BMI (numerical) | 1.02 | 0.94–1.1 | 0.654 |
| Obesity | 1.71 | 0.59–4.41 | 0.287 |
| Age (numerical) | 0.97 | 0.94–1 | 0.0476 |
| Leukemia subtype | ALL: reference  AML: 0.1  APL: 0.23 | 0.02–0.32  0.05–0.72 | <0.001  0.023 |
| White blood cell count (x10^9^/L) | 1 | 1–1 | 0.775 |
| Peripheral blasts (%) | 0.99 | 0.98–1.01 | 0.439 |
| Peripheral blood monocytes (%) | 1 | 1–1 | 0.665 |
| Platelets ≥20x10^9^/L | 2.9 | 0.97–12.63 | 0.09 |
| Prothrombine time ratio | 1.05 | 0.58–1.35 | 0.756 |
| CRP | 1 | 1–1.01 | 0.695 |
| Use of asparaginase  (ALL only) | 1.1 | 0.35–3.48 | 0.869 |
| Intensive treatment | 1.01 | 0.05–5.26 | 0.994 |

*OR: Odds-ratio; CI: confidence interval; BMI: body mass index; CRP: C-reactive protein.

***Table S3:*** *Univariate analysis of predictive scores for venous thrombosis (VT) (excluding superficial events and catheter-related thrombosis).*

|  | **Without TE** | **With TE** | **p-value** |
| --- | --- | --- | --- |
| **Khorana Score (N=327)** | | | 0.772 |
| Low | 16 (5.2%) | 1 (4.8%) |  |
| Intermediate | 285 (92.5%) | 19 (90.5%) |  |
| High | 7 (2.3%) | 1 (4.8%) |  |
| **DIC Score ≥ 5 (N=227)** | | | 1.000 |
|  | 186 (87.3%) | 12 (85.7%) |  |
| **Median DIC (Mean [SD])** **(N=227)** | | | 0.540 |
|  | 5.77 (1.33) | 6 (1.36) |  |
| **SiAML high risk (N=246)** | | | 1.000 |
|  | 17 (7.4%) | 1 (6.7%) |  |

*DIC: Disseminated intravascular coagulation.

***Table S4:*** *Univariate analysis of predictive scores for for thrombotic events (TE) at 60 days in ALL patients.*

| Variable | OR | CI 95% | p-value |
| --- | --- | --- | --- |
| Male sex | 0.56 | 0.23–1.37 | 0.205 |
| BMI (numerical) | 0.99 | 0.9–1.07 | 0.748 |
| Obesity | 1.9 | 0.65–5.18 | 0.221 |
| Age (numerical) | 0.98 | 0.96–1.01 | 0.224 |
| White blood cell count (x10^9^/L) | 1 | 1–1 | 0.513 |
| Fibrinogen | 1 | 1–1 | 0.323 |
| D-dimmer | 1 | 1–1 | 0.712 |
| CNS disease | 0.7 | 0.15–2.42 | 0.608 |
| Peripheral blasts (%) | 0.99 | 0.98–1.01 | 0.356 |
| Peripheral blood monocytes (%) | 1 | 1–1 | 0.178 |
| Platelets ≥20x10^9^/L | 4.28 | 1.36–18.99 | 0.0256 |
| Prothrombine time ratio | 0.85 | 0.14–1.33 | 0.688 |
| CRP | 1 | 0.99–1.01 | 0.815 |
| Use of asparaginase | 1.11 | 0.43–2.9 | 0.828 |
| Intensive treatment | 0.6 | 0.09–2.42 | 0.525 |

***Table S5:*** *Univariate analysis of predictive scores for for thrombotic events (TE) at 60 days in AML patients.*

| Variable | OR | CI 95% | p-value |
| --- | --- | --- | --- |
| Male sex | 1.54 | 0.64–3.86 | 0.341 |
| BMI (numerical) | 1.02 | 0.93–1.12 | 0.666 |
| Obesity | 1.33 | 0.35–4.17 | 0.641 |
| Age (numerical) | 1.02 | 0.99–1.06 | 0.184 |
| White blood cell count (x10^9^/L) | 1 | 1–1 | 0.162 |
| Fibrinogen | 1 | 1–1 | 0.293 |
| D-dimmer | 1 | 1–1 | 0.765 |
| Peripheral blasts (%) | 1.02 | 1–1.03 | 0.0244 |
| Peripheral blood monocytes (%) | 0.99 | 0.98–1 | 0.23 |
| Platelets ≥20x10^9^/L | 1.74 | 0.62–6.21 | 0.337 |
| Prothrombine time ratio | 0.85 | 0.14–1.33 | 0.688 |
| CRP | 1 | 1–1.01 | 0.0506 |
| Intensive treatment | 1.62 | 0.08–10.65 | 0.667 |
